# Tracking the molecular evolution and transmission patterns of SARS-CoV-2 lineage B.1.466.2 in Indonesia based on genomic surveillance data

**DOI:** 10.1186/s12985-022-01830-1

**Published:** 2022-06-16

**Authors:** Mingjian Zhu, Qianli Zeng, Bryanna Infinita Laviashna Saputro, Sien Ping Chew, Ian Chew, Holie Frendy, Joanna Weihui Tan, Lanjuan Li

**Affiliations:** 1 State Key Laboratory for Diagnosis and Treatment of Infectious Diseases, National Clinical Research Center for Infectious Diseases, National Medical Center for Infectious Diseases, Collaborative Innovation Center for Diagnosis and Treatment of Infectious Diseases, The First Affiliated Hospital, Zhejiang University School of Medicine, Hangzhou, China; 2 Shanghai Institute of Biological Products, Shanghai, China; 3 Faculty of Medicine, University of Indonesia, Jakarta, Indonesia; 4 Shanghai Jiao Tong University School of Medicine, Shanghai, China; 5 Zhejiang University School of Medicine, Hangzhou, China; 6 Faculty of Medicine and Health Sciences, Krida Wacana Christian University, Jakarta, Indonesia; 7 Faculty of Arts and Social Sciences, National University of Singapore, Singapore, Singapore

**Keywords:** Severe acute respiratory syndrome coronavirus 2, B.1.466.2 variant, Genomic surveillance, Prevalence, Phylogenesis, Single nucleotide variant, Effective population size, Effective reproduction number, Transmission patterns, Indonesia

## Abstract

**Background:**

As a new epi-center of COVID-19 in Asia and a densely populated developing country, Indonesia is facing unprecedented challenges in public health. SARS-CoV-2 lineage B.1.466.2 was reported to be an indigenous dominant strain in Indonesia (once second only to the Delta variant). However, it remains unclear how this variant evolved and spread within such an archipelagic nation.

**Methods:**

For statistical description, the spatiotemporal distributions of the B.1.466.2 variant were plotted using the publicly accessible metadata in GISAID. A total of 1302 complete genome sequences of Indonesian B.1.466.2 strains with high coverage were downloaded from the GISAID’s EpiCoV database on 28 August 2021. To determine the molecular evolutionary characteristics, we performed a time-scaled phylogenetic analysis using the maximum likelihood algorithm and called the single nucleotide variants taking the Wuhan-Hu-1 sequence as reference. To investigate the spatiotemporal transmission patterns, we estimated two dynamic parameters (effective population size and effective reproduction number) and reconstructed the phylogeography among different islands.

**Results:**

As of the end of August 2021, nearly 85% of the global SARS-CoV-2 lineage B.1.466.2 sequences (including the first one) were obtained from Indonesia. This variant was estimated to account for over 50% of Indonesia’s daily infections during the period of March–May 2021. The time-scaled phylogeny suggested that SARS-CoV-2 lineage B.1.466.2 circulating in Indonesia might have originated from Java Island in mid-June 2020 and had evolved into two disproportional and distinct sub-lineages. High-frequency non-synonymous mutations were mostly found in the spike and NSP3; the S-D614G/N439K/P681R co-mutations were identified in its larger sub-lineage. The demographic history was inferred to have experienced four phases, with an exponential growth from October 2020 to February 2021. The effective reproduction number was estimated to have reached its peak (11.18) in late December 2020 and dropped to be less than one after early May 2021. The relevant phylogeography showed that Java and Sumatra might successively act as epi-centers and form a stable transmission loop. Additionally, several long-distance transmission links across seas were revealed.

**Conclusions:**

SARS-CoV-2 variants circulating in the tropical archipelago may follow unique patterns of evolution and transmission. Continuous, extensive and targeted genomic surveillance is essential.

**Supplementary Information:**

The online version contains supplementary material available at 10.1186/s12985-022-01830-1.

## Background

Coronavirus disease 2019 (COVID-19) is an infectious disease caused by a novel β-coronavirus, namely, severe acute respiratory syndrome coronavirus 2 (SARS-CoV-2) [1, 2]. It has evolved into an ongoing pandemic since 11 March 2020 [3, 4]. According to the World Health Organization (WHO), over 215 million confirmed cases with nearly 4.5 million related deaths have been reported globally as of the end of August 2021 [5]. Straddling Southeast Asia and Oceania, Indonesia is the world's largest archipelago nation and the world’s fourth most populous nation. Despite some degree of underestimation, Indonesia has been bearing the highest cumulative burden of COVID-19 in the region of the Association of Southeast Asian Nations (ASEAN) since mid-October 2020 [6]. As of 6 June 2021, this populous tropical country had only 5.51% of its citizens fully vaccinated and was once considered to be Asia’s new epi-center of the pandemic after India [7, 8].

With the worldwide spread of the virus, its continuous evolution yielded the emergence of multiple variants, which has posed an increased risk to global public health [9]. In this context, sequencing-based genomic surveillance, whether at the national, regional or global level, has become the key to formulating effective intervention strategies [10]. The Pango nomenclature offers a dynamic proposal for the classification and subtyping of SARS-CoV-2 strains based on genetic relatedness [11]. Under this system, a multitude of SARS-CoV-2 lineages and sub-lineages are identified and named accurately, including four variants of concern (VOCs: B.1.1.7, B.1.351, P.1 and B.1.617.2 corresponding to Alpha, Beta, Gamma and Delta labelled by WHO) and several variants of interest (VOIs) [12]. Since 2020, several teams and institutions (e. g., the Yogyakarta-Central Java COVID-19 Study Group and the West Java Health Laboratory) have conducted whole-genome sequencing (WGS) of SARS-CoV-2 strains circulating in various parts of Indonesia, and have shared genetic data and related metadata via the Global Initiative on Sharing Avian Influenza Data (GISAID) platform [13–17]. These efforts make it possible to gain insights into Indonesia's COVID-19 epidemic from a micro perspective.

One of the WHO-designated variants under monitoring (VUMs), SARS-CoV-2 lineage B.1.466.2, was first documented in Indonesia in November 2020 and was reported to contribute to 31.7% of all cases nationwide as of the third week of August 2021 (second only to the Delta variant) [12, 18]. During the pre-Delta outbreak period, the B.1.466.2 lineage was the most dominant variant in Indonesia whereas the Alpha variant was more dominating in other parts of Asia [19]. Although the B.1.466.2 variant has been introduced into neighbouring countries (e. g., Malaysia) [20], it still mainly circulates in Indonesia and might have accumulated unique mutations in indigenous transmission. Due to its overly dispersed territory and relatively limited health resources, extensive and continuous SARS-CoV-2 genomic surveillance remains a daunting task for Indonesia [21]. Two bioinformatic analyses of SARS-CoV-2 full-length sequences from Indonesia confirmed the dominance of GH clade with marker mutations of D614G and Q57H [22, 23]. A more representative recent study found that in Indonesia, the B.1.466.2 variant shared some missense mutations with both Alpha and Delta VOCs, indicating a significant impact on transmission and clinical severity; the N439K mutation showing antibody resistance was prevalent in the Indonesian B.1.466.2 variant [19]. Other related studies mainly focused on a certain province or part of Indonesia (especially the Java Island) [13–17]. In West Java, the B.1.466.2 variant dominated the local SARS-CoV-2 infections from January through April 2021, which was generally consistent with the frequency trend of Q57H mutation [17]. In Central Java and Yogyakarta, around 13% of SARS-CoV-2 samples (collected from May 2020 to June 2021) were clustered into the B.1.466.2 lineage, ranking second among non-Delta variants [16]. As an important local variant, there is a lack of nationwide genomic surveillance targeting the B.1.466.2 lineage. In particular, the trajectory of its population size over time and the picture of how it transmitted between different islands in Indonesia remain unclear.

In the present study, we retrospectively analyzed the whole-genome sequencing data of SARS-CoV-2 lineage B.1.466.2 from Indonesia to systematically reveal its evolutionary features and transmission patterns in such a tropical archipelago setting.

## Methods

### Study area

Indonesia is divided into 34 first-level administrative regions which are located on five main islands (Java, Sumatra, Kalimantan, Sulawesi and Papua) and two main archipelagos (Nusa Tenggara and Maluku). Geographically, Bali is the westernmost part of Nusa Tenggara but it is traditionally listed as a separate unit in the statistics. Following the Wallace Line in evolutionary ecology, Java, Bali, Sumatra and Kalimantan belong to Western Indonesia, while Sulawesi, Nusa Tenggara, Maluku and Papua belong to Eastern Indonesia. Western Indonesia has a larger population and a higher level of socio-economic development than Eastern Indonesia [24].

### Data collection

All SARS-CoV-2 genomic data in the study were downloaded from the GISAID’s EpiCoV database (https://www.gisaid.org/) on 28 August 2021. The relevant retrieval fields were set as follows: (1) “hCoV-19” as virus name; (2) “Asia/Indonesia” as location; (3) “Human” as host. For quality control, sequences that meet any of the following filtering criteria should be excluded: (1) length < 29000nt; (2) proportion of undetermined nucleotide bases > 5%; (3) Vero cell passage history; (4) incomplete collection date; (5) unknown Pango lineage. Finally, a total of 4210 complete and high-coverage SARS-CoV-2 genomes collected from Indonesia between 12 March 2020 and 17 August 2021 were included and compiled into Dataset 1. Based on Dataset 1, 1302 genomes belonging to the B.1.466.2 lineage (with the collection date ranging from 6 August 2020 to 2 August 2021) were extracted to constitute Dataset 2 for further analysis. The accession IDs for relevant sequences are available in Additional file 1.

### Statistics on lineage prevalence

The global epidemic burden of the SARS-CoV-2 lineage B.1.466.2 was characterized by the national distribution of genomic samples, which can be directly retrieved in GISAID. Using the metadata in Dataset 2, we calculated the absolute counts and proportions of the B.1.466.2 samples according to the eight regions of Indonesia mentioned above. In time series, the daily sampling counts of the Indonesian B.1.466.2 variant were smoothed by the seven-day rolling average method. The dynamic proportions of the B.1.466.2 and Delta (lineage B.1.617.2 and its sub-lineages) variants among SARS-CoV-2 infections in Indonesia were estimated with the genomic data in Dataset 1 using the R “ratesci” package v0.3-0 (Peter J. Laud, Statistical Services Unit, University of Sheffield, Sheffield, United Kingdom); the corresponding 95% confidence intervals were calculated based on Bayesian Jeffrey’s prior [25].

### Genome-wide phylogenetic and mutation analyses

The 1302 full‐length genome sequences in Dataset 2 were aligned using MAFFT v7.487 (Osaka University, Suita, Osaka, Japan). We performed the phylogenetic analysis of Indonesian SARS-CoV-2 lineage B.1.466.2 strains with maximum likelihood (ML) method based on the general time reversible model [26]. The ML phylogenetic tree was built using FastTree v2.1.10 (Morgan N. Price, Physical Biosciences Division, Lawrence Berkeley National Laboratory, CA, USA) [27], and the branches with low support (bootstrap value < 0.7) were removed. A root-to-tip regression was conducted to test the temporal signal in Dataset 2 using TreeTime v0.8.4 (Pavel Sagulenko, Emma Hodcroft & Richard Neher, Max Planck Institute for Developmental Biology, Tubingen, Germany) [28], and the effect was evaluated with R square calculated using TempEst v1.5.3 [29]. Based on the ML phylogeny and temporal structure, the corresponding time-scaled phylogenetic tree was built with the "least square" re-rooting scheme. The “Mugration” model in TreeTime v0.8.4 was applied to estimate the discrete state (region) and its probability for each most recent common ancestor (MRCA) of the time-scaled phylogenetic tree [30]. Taking Wuhan-Hu-1 (NC_045512.2) as the reference genome, single nucleotide variants (SNVs) were called and annotated using the Nextclade tool (https://clades.nextstrain.org/) [31]. The time-scaled phylogenetic tree and the SNV matrix corresponding to the leaf nodes were visualized using the iTOL tool (https://itol.embl.de/) [32].

### Phylodynamic and phylogeographic reconstructions

Using TreeTime v0.8.4, the maximum likelihood approach was applied to estimate the effective population size (Ne) of SARS-CoV-2 lineage B.1.466.2 circulating in Indonesia over time [28]. According to the dynamics of Ne, four phases were determined for better understanding the demographic history of this local variant. As an indicator of real-time virus transmissibility, the effective reproduction number (Rt) was estimated using the R (R Foundation for Statistical Computing, Vienna, Austria) “EpiEstim” package v2.2-4 based on the time series of daily sampling counts [33].

To reconstruct the phylogeography of the B.1.466.2 variant within Indonesia, the coalescent events from the time-scaled phylogenetic tree were used to represent the transmission events. After filtering out the clades with MRCA discrete state probability less than 0.7, the transmission events were counted by region in each phase [34]. For a given region in Indonesia, the level of local transmission was evaluated with the ratio of inferred intra-regional transmission events to inter-regional transmission events (including importations and exportations) [35]. A series of maps were made to visualize the inferred transmission patterns of SARS-CoV-2 lineage B.1.466.2 in Indonesia during different phases using the R “Leaflet” package v2.0.4.1 [36].

## Results

### Spatiotemporal distribution characteristics

**Fig. 1:**
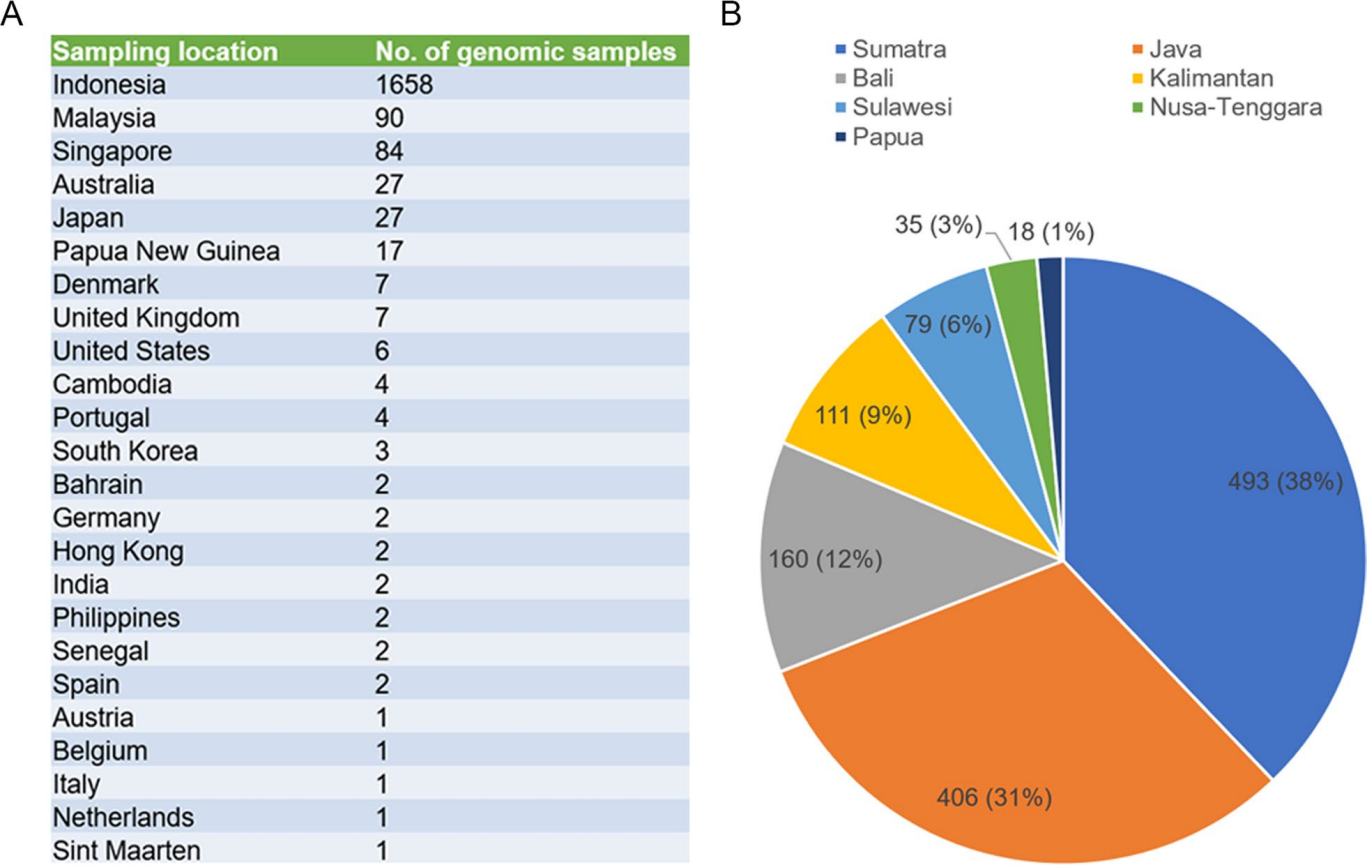
**A** Table displaying the global distribution of SARS-CoV-2 lineage B.1.466.2. **B** Pie chart displaying the geographical distribution of SARS-CoV-2 lineage B.1.466.2 in Indonesia; numbers of genomic samples from different regions and corresponding percentages are labeled (no samples from Maluku). Both statistics are based on genome samples recorded in GISAID as of the end of August 2021

As of 28 August 2021, the GISAID database recorded a total of 1953 complete genomes of SARS-CoV-2 lineage B.1.466.2 from 24 countries. Of these, 84.9% were from Indonesia, followed by Malaysia (4.6%), Singapore (4.3%), Australia (1.4%), Japan (1.4%) and Papua New Guinea (0.9%) (Fig. 1A). The first sample (accession ID: EPI_ISL_2868921) was isolated from Banten Province (a part of Java), Indonesia on 6 August 2020. The geographic distribution of 1302 Indonesian SARS-CoV-2 lineage B.1.466.2 samples included in Dataset 2 is shown in Fig. 1B. There were 493 samples from Sumatra and 406 samples from Java, together accounting for 69.0%. There were no samples from Maluku.

**Fig. 2:**
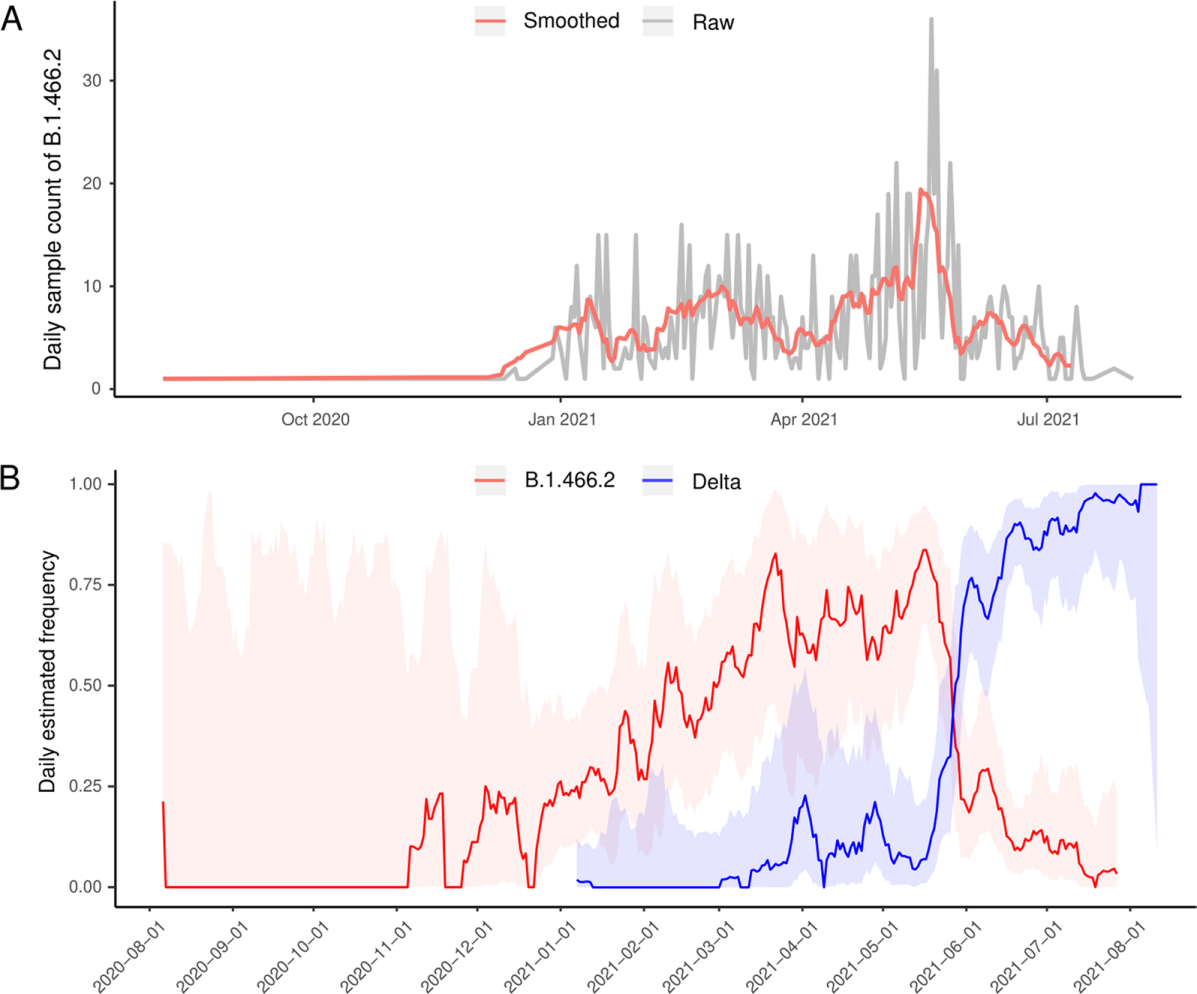
**A** Time series of daily sampling counts of the Indonesian SARS-CoV-2 B.1.466.2 variant expressed with raw data (grey line) and seven-day rolling averages (red line). **B** Estimated proportion dynamics of the B.1.466.2 and Delta (lineage B.1.617.2 and its sub-lineages) variants in Indonesia; the colored bands represent corresponding 95% confidence intervals

The daily count of Indonesian SARS-CoV-2 lineage B.1.466.2 samples began to increase significantly in early January 2021 and reached a peak in late May 2021 (36 sequences collected on 19 May 2021), and rapidly decreased thereafter (Fig. 2A). Among 4210 samples of various SARS-CoV-2 variants from Indonesia(Dataset 1), the B.1.466.2 variant was the dominant one from early March 2021 to late May 2021 and reached its peak proportion (83.7%) with a 95% confidence interval (95% CI) of 65.5% ~ 94.8% on 16 May 2021 (Fig. 2B). Contrary to the B.1.466.2 variant, the Delta variant experienced a sharp increase in its proportion during May 2021, making it the latest dominant variant in Indonesia.

### Time-scaled phylogeny and mutation profiles

**Fig. 3:**
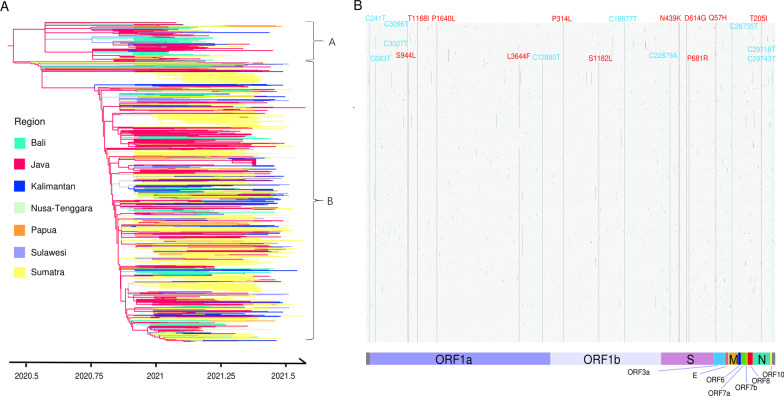
**A** Time-scaled maximum-likelihood phylogenetic tree of SARS-CoV-2 lineage B.1.466.2 circulating in Indonesia; colors indicate different sampling locations. **B** Genome-wide single nucleotide variation (SNV) matrix of the Indonesian SARS-CoV-2 B.1.466.2 variant (against the reference genome); major non-synonymous mutations are marked in red, while major synonymous mutations are marked in blue

As shown in Additional file 2, a positive temporal signal in Dataset 2 was detected using root-to-tip regression analysis (R^2^ = 0.362; *P* < 0.01). The evolutionary rate of this lineage in Indonesia was roughly estimated at 7.446 × 10^−4^ substitutions per site per year. Furthermore, the time-scaled phylogenetic relationships of SARS-CoV-2 B.1.466.2 variants from Indonesia were reconstructed using maximum-likelihood phylodynamic analysis (Fig. 3A). The root of the evolutionary tree was located in Java (bootstrap support: 99.3%), and the time to the MRCA was dated to 13 June 2020.

According to the tree topology, two sub-lineages (labeled A and B) were identified, with 161 (12.4%) and 1141 (87.6%) samples respectively. The samples from Java were relatively evenly distributed in clades, while over 95.0% of those from Sumatra and Kalimantan were clustered in the B sub-lineage. The common amino acid changes (with mutation frequencies > 99%) of the B.1.466.2 variant circulating in Indonesia included: ORF1a-T1168I, ORF1a-P1640L, ORF1b-P314L, S-N439K, S-D614G, ORF3a-Q57H and N-T205I (Fig. 3B). Compared with sub-lineage A, sub-lineage B was additionally characterized by amino acid changes such as ORF1a-S944L, ORF1a-L3644F, ORF1b-S1182L and S-P681R.

### Reconstructed demographic history and epidemic dynamics

**Fig. 4:**
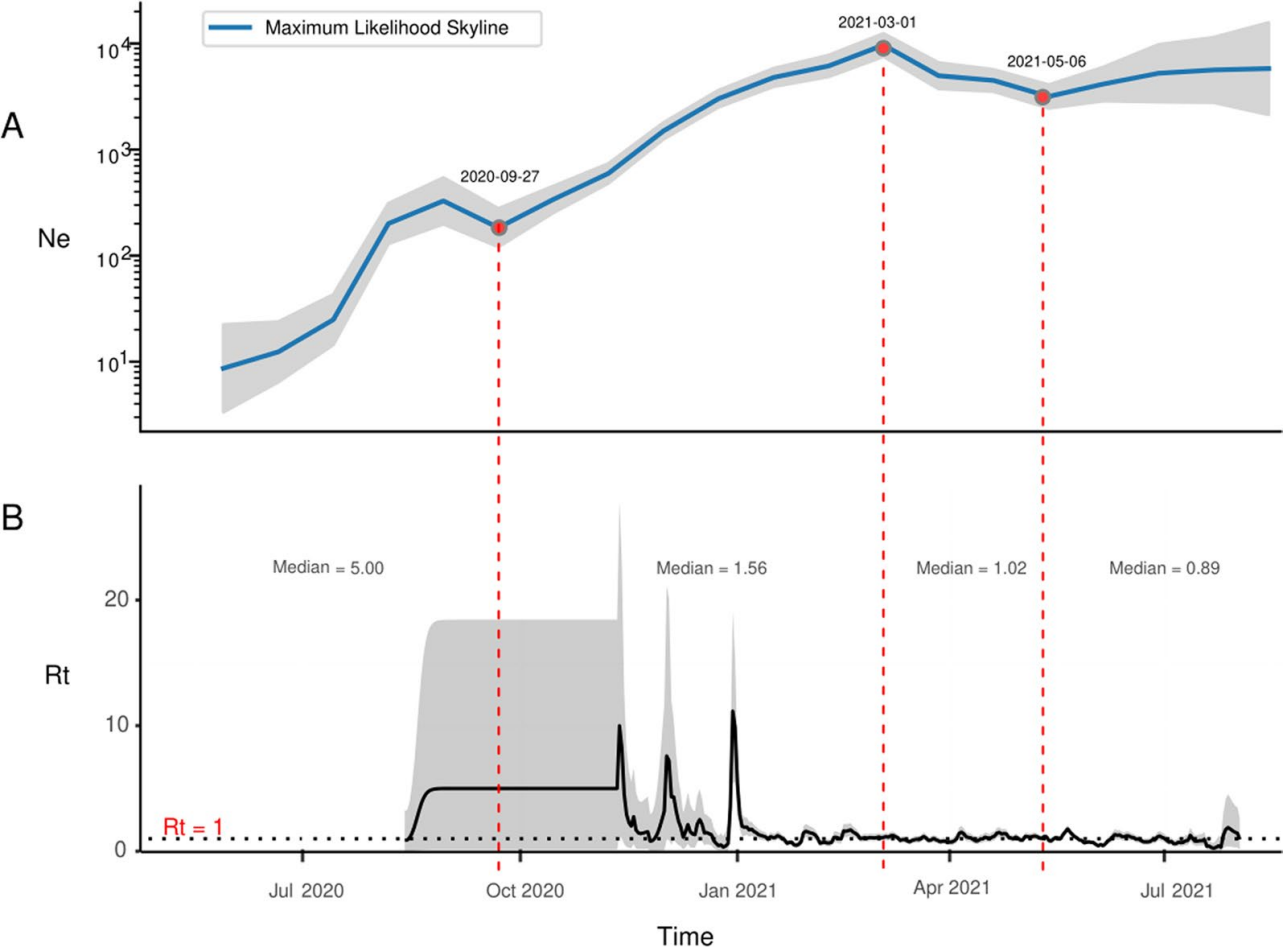
**A** Maximum likelihood skyline displaying the effective population size (Ne) trajectory of SARS-CoV-2 lineage B.1.466.2 circulating in Indonesia; the blue line represents the point estimates for Ne, while the red dots represent the cut-off points of phase division. **B** Estimated effective reproduction number (Rt) dynamics of the Indonesian SARS-CoV-2 B.1.466.2 variant; the median Rt estimates for the four phases (divided according to the Ne trajectory) are indicated. The grey bands represent corresponding 95% confidence intervals

The historical trajectory of effective population size of SARS-CoV-2 lineage B.1.466.2 circulating in Indonesia could be divided into four phases (Fig. 4A). From mid-June 2020 to late September 2020, the Ne rose in volatility based on an initial low level. During the five months after the end of September 2020, this lineage underwent a steady exponential expansion of the viral population in Indonesia; the Ne was estimated to multiply 50-fold and reach its peak (9.7 × 10^3^) at the beginning of March 2021. In the third phase (early March to early May, 2021), a slight population contraction of the variant was inferred. Finally, the viral population seemed to remain relatively steady with a mean Ne of 4.8 × 10^3^.

The effective reproduction number of SARS-CoV-2 lineage B.1.466.2 in Indonesia between early August 2020 and early August 2021 was estimated using information from 1302 complete genomes (Fig. 4B). During the investigation period of one year, Rt showed an overall downward trend, but showed different characteristics in four phases. The median estimate of Rt for the first phase was the highest (5.00), but its confidence interval was wide due to the limited sample size. Three Rt peaks were observed in the second phase, and the highest one reached 11.18 (95% CI: 5.36 ~ 19.10) on 23 December 2020. During the third phase, the estimated Rt remained relatively stable around the cut-off value of one. The median Rt for the last phase was estimated to be 0.89 (< 1.00).

### Spatiotemporal transmission patterns

**Fig. 5:**
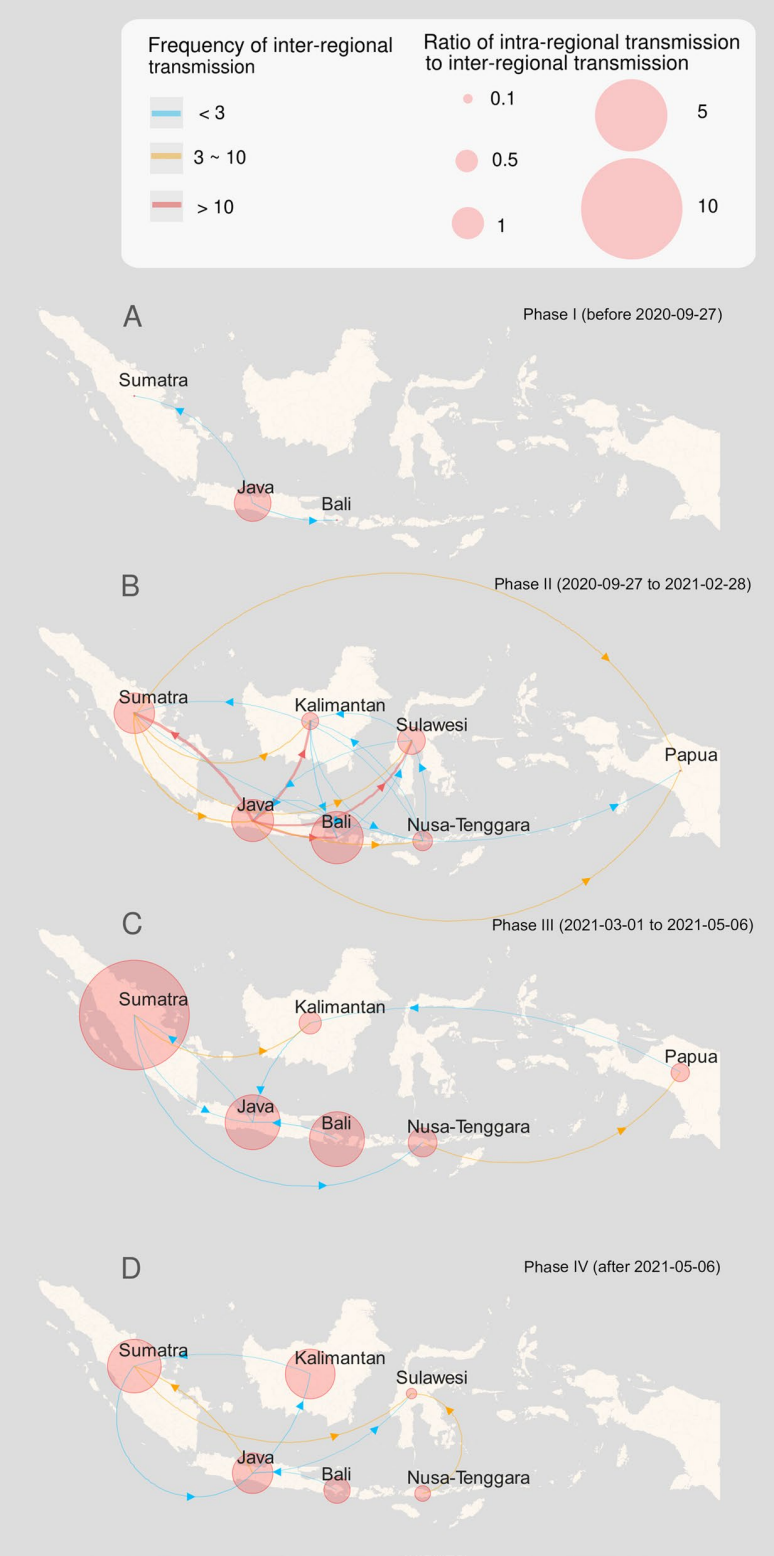
A series of maps showing the inferred transmission of the SARS-CoV-2 B.1.466.2 variant in Indonesia’s different regions in each phase. Panels** A**,** B**,** C** and** D** correspond to Phases I, II, III and IV respectively. Line colors indicate the inter-regional transmission intensity, while the circle size indicates the relative strength of spread within the region

The inferred transmission of the B.1.466.2 variant between different regions of Indonesia in each phase was mapped. By the end of September 2020, the scope of transmission was limited, presenting the epi-center in Java and spilling to neighboring Sumatra and Bali (Fig. 5A). The second phase (October 2020 to February 2021) was inferred to be a critical period for the B.1.466.2 prevalence in Indonesia, both in terms of spread coverage and transmission intensity (Fig. 5B). Java exported this variant at a higher intensity to all other regions and constituted bidirectional transmission loops with Sumatra, Bali and Sulawesi, respectively. There was a long-distance transmission link from Sumatra to Papua. Intra-regional transmission occurred in all regions except Papua, especially in Java, Sumatra and Bali. During the following two months, inter-regional transmission became sparse and weak (Fig. 5C). The transmission loop between Java and Sumatra still existed, and Sumatra acted as an emerging epi-center with upgraded intra-regional transmission. After early May 2021, inter-regional transmission remained relatively low, characterized by viral introductions from Sumatra, Java and Nusa Tenggara to Sulawesi, as well as bidirectional viral exchanges between Sumatra and Java (Fig. 5D). The intra-regional transmission in Kalimantan increased while those in previous hotspots declined. In general, the prevalence of SARS-CoV-2 lineage B.1.466.2 in Indonesia followed spatiotemporal patterns of strong in the western part and weak in the eastern part, rising in the early stage and declining in the later stage. The inferred frequencies for both intra- and inter-regional transmission events are given in Additional file 3.

## Discussion

As the COVID-19 pandemic continues to progress globally, the evolution and transmission dynamics of various SARS-CoV-2 variants have aroused great interest among virologists, clinicians, epidemiologists, and even health decision-makers. This study revealed the prevalence, evolution and spatiotemporal transmission of an endemic variant (the lineage B.1.466.2) in Indonesia using publicly available complete genomes and associated metadata. Such findings provide valuable reference for responding to future outbreaks in archipelagic countries.

More than 80% of the SARS-CoV-2 lineage B.1.466.2 genomes are from Indonesia, and other countries with a contribution rate of more than 1% (except Japan) are adjacent to Indonesia. The geographic distribution supported the local prevalence of this variant with the Indonesian Archipelago as its global epi-center [17]. Furthermore, the number of samples contributed by each region in Indonesia was generally consistent with its population size and laboratory diagnostic capacity [21]. It should be pointed out that sampling may have been biased by the availability of sequencing centers and resources, which was relatively low in Eastern Indonesia (especially before the establishment of the nationwide network for SARS-CoV-2 genomic surveillance) [37]. The B.1.466.2 variant was first documented in Indonesia in November 2020 [12]. However, the collection time of one Indonesian B.1.466.2 sample submitted to GISAID can be traced back to 6 August 2020. Hence, we inferred that this variant was likely to have been circulating in Indonesia before August 2020. In the study, we observed a noticeable shift in the dominant strain of Indonesian SARS-CoV-2 infections, which was from the B.1.466.2 variant to the B.1.617.2 or Delta variant and took place in late May 2021. Interestingly, the prevalence of these two variants in Indonesia was almost complementary in time series. This phenomenon reflected the fierce competition between different variant populations of the same virus, and in this case, the highly contagious Delta variant eventually gained a competitive advantage [38]. As the virus continues to evolve, similar population succession is likely to be repeated, which calls for real-time genomic surveillance [10].

The time-scaled phylogeny revealed the most recent common ancestor and heterogeneous evolution of this lineage. According to the root state in the evolutionary tree, the B.1.466.2 variant circulating in Indonesia was estimated to have originated in Java in mid-June 2020. The estimate of its MRCA was logically consistent with the sampling location and date of the earliest B.1.466.2 sequence. To comprehend the emergence and evolution of pathogenic viruses, the complex "host–pathogen-environment" interaction provides an analytical framework [39]. As the world's most populous island, Java is home to 147.8 million people (over 50% of the Indonesia’s total population) [40]. Java's very high population density (~ 1200/km^2^) means a high SARS-CoV-2 transmission rate [41], superimposed on its large number of potential hosts, which can lead to large viral population size and intense selection pressure—two major evolutionary drivers [42]. Therefore, it seemed unsurprising that this local variant of Indonesia originated from Java. In addition to the root, the trunks to which side branches belong were also assigned to Java, indicating that the SARS-CoV-2 lineage B.1.466.2 strains in other parts of Indonesia were all introduced from Java. Like other forms of life on earth, the evolution of viruses is subjected to environmental selection. Among the side branches of the evolutionary tree, we observed the clustering preference of sequences derived from Sumatra and Kalimantan in the B clade (sub-lineage). The equator traverses Sumatra and Kalimantan, and both large islands are known for their dense jungles and diverse species. At least 88 and 97 species of tropical bats have been identified in Sumatra and Kalimantan respectively, including those that potentially serve as natural hosts for SARS-CoV-2 [43–45]. Interestingly, a viral ecology study in the islands of the Western Indian Ocean found a strong signal of co-evolution between coronaviruses and their bat host species and further revealed the effect of the endemism resulted from geographical isolation on viral diversification within bat families [46]. Therefore, we speculate that the unique jungle ecology of Sumatra and Kalimantan may contribute to the local circulation of the B.1.466.2 variant through certain species of bats (e. g. *Rhinolophus* spp.) [47, 48].

The evolutionary rate of Southeast Asian SARS-CoV-2 strains (isolated before September 2020) was estimated to be 1.446 × 10^−3^ substitutions per site per year [49], while the corresponding estimate for the Indonesian B.1.466.2 variant was almost halved. The decline in the rate of evolution probably occurred under its RNA proofreading mechanism and partly reflected that this variant was more adaptable to the local environment than the original strain [50, 51]. Several high-frequency non-synonymous mutations were identified in the SARS-CoV-2 lineage B.1.466.2 from Indonesia, most of which occurred in the non-structural protein 3 (NSP3) and the spike protein. NSP3 is the largest viral protein containing multiple domains and participates in the formation of the replication/transcription complex [52]**.** Notably, the S944L mutation (only observed in the B sub-lineage) is located in the papain-like protease domain of NSP3. However, whether this mutation can improve viral fitness remains to be verified in further experiments [53]. Co-mutations of S-D614G and ORF3a-Q57H are the genetic markers for the GH clade, to which the Pango lineage B.1.466.2 belongs according to GISAID [54]. The D614G mutation in the spike protein confers a selective advantage on SARS-CoV-2 and eventuates in all VOIs and VOCs [55]. Previous studies have proven that the D614G strain has higher infectivity, primarily by altering the conformation of the RBD (receptor binding domain) and enhancing the affinity with the ACE2 (angiotensin-converting enzyme 2) receptor [56–58]. The Q57H mutation causes major truncation of ORF3b and structural instability of ORF3a [59]. SARS-CoV-2 strains with this mutation are reported to evade innate immune activation and inflammatory responses [60, 61]. As the second most common RBD mutation worldwide, the S-N439K mutation has been shown not only to enhance the infectivity of the virus (probably via creating a new salt bridge with ACE2), but also to increase the resistance to the neutralizing antibody [62, 63]. Located in the furin cleavage site of the spike protein, the P681R mutation is one of the defining mutations for the current dominant strain globally—the Delta variant [64]. It was reported that this highly conserved mutation could facilitate furin-mediated S1–S2 cleavage, accelerate membrane fusion and internalization, enhance replication efficiency and promote resistance to neutralizing antibodies [65–67]. In this study, we found that the sub-lineage B of the Indonesian B.1.466.2 variant has evolved the S-P681R mutation, and the numerical advantage of the B sub-lineage over the A lineage (approximately 7:1) supported the potential higher infectivity of the strain with this mutation. Although the effect of a single amino acid mutation on viral infectivity, virulence and immunological properties is generally limited, the accumulation of some significant mutations in a background of multiple mutations may profoundly alter the pandemic patterns and the clinical outcomes, as we have seen with the Delta variant [15, 16, 68]. Given the mutation profiles observed (especially the D614G/N439K/P681R co-mutations in the spike protein), continuous monitoring of the B.1.466.2 variant remains essential.

In epidemiological contexts, Ne (a measure of genetic diversity) is generally considered to be proportional to the number of infected individuals [69]. For the COVID-19 pandemic, Ne may reflect the incidence of SARS-CoV-2 infection among the population due to the short transmission window of the virus [70]. Considering the decline in susceptible individuals and the implementation of intervention strategies, Rt is commonly used to assess the real-time dynamics of infectious disease transmission [71]. In the initial phase after the emergence of the B.1.466.2 variant, its effective population expanded the fastest and corresponded to a very high Rt, suggesting that the local hosts were highly susceptible to the new variant. An exponential growth in Ne with a median Rt > 1 was observed during October 2020–February 2021, which documented the widespread of SARS-CoV-2 lineage B.1.466.2 in communities (especially in the form of infection clusters). Moreover, the peak of estimated Rt overlapped with the peak of total reported cases nationwide in January 2021 [72], indicating that the B.1.466.2 variant could be responsible for the surge of total SARS-CoV-2 infections at the period. From early March 2021 to early May 2021, the Ne of the B.1.466.2 variant showed a decreasing trend, which may have resulted from the competitive pressure brought by the Delta variant, the enforcement of community-level public activity restrictions (locally known as PPKM) and the launching of Indonesian nationwide vaccination program against COVID-19 [73]. Similar reasons could be used to explain the lower Rt. In the final phase of the study, the median Rt estimate < 1 and the plateaued Ne suggested that SARS-CoV-2 lineage B.1.466.2 seemed to be gradually extinct in Indonesia. However, in this case we could not exclude the possibility that the B.1.466.2 variant was still circulating in certain regions due to the lag in SARS-CoV-2 genome submissions to GISAID [74]. Similar to some of its relatives (e. g., OC43 and HKU1), the circulation pattern of SARS-CoV-2 also exhibits a certain seasonality [75, 76]. During the period of December 2021 to March 2022 (just in another rainy season), the B.1.466.2 variant was detected again in several regions of Indonesia [77], which may in part reflect seasonal outbreaks.

We assessed the inter-regional transmission and the relative advantage of intra-regional transmission over inter-regional transmission of SARS-CoV-2 lineage B.1.466.2 in Indonesia in the four phases (divided based on the inferred Ne trajectory). Java was estimated to be the initial epi-center for this SARS-CoV-2 variant, which was consistent with the estimated origin located in Java. In addition, a certain degree of intra-regional transmission was likely to have already existed in Java before the first infection with this variant was officially documented. This finding highlighted the importance of improved genomic surveillance for the timely identification of new SARS-CoV-2 variants. Corresponding to the exponential growth of Ne, the transmission of the B.1.466.2 variant in Indonesia reached its peak during the second phase, which coincided with the country’s rainy season (October to March) and several important festivals. The rainy season usually means more water vapour in air and less ultraviolet radiation from sunlight. In flow physics, higher ambient relative humidity contributes to longer lifetime of respiratory droplets [78]. Previous studies demonstrated that ultraviolet light has the strongest correlation with lower COVID-19 growth [76, 79, 80], probably due to the inactivation of the virus by ultraviolet light and its promotion of the vitamin D (as an immune regulator) synthesis. In addition, Indonesia is a country prone to flooding, and serious flood disasters mostly occur in the rainy season every year and mainly affect populated areas like Java and Sumatra [81]. Flooding can cause both difficulties in practicing social distancing during evacuation and interruptions in the supply of medical protective materials, facilitating the local transmission of the virus [82, 83]. Thus, strengthening the prevention, preparedness and readiness for COVID-19 in the rainy season is needed. A large number of studies have confirmed the contribution of population mobility to the spread of COVID-19 [84–86]. To transition to a healthy, safe and productive society, the Indonesian government changed the strictest intervention policy of large-scale social restrictions (PSBB) into the “PSBB transisi” policy [87]. This allowed some citizens (especially those living in the capital, Jakarta) to return home to visit their relatives during Christmas 2020 and the following New Year holiday, partly contributing to the spread across regions [88]. The sea barrier makes inter-regional passenger traffic in Indonesia largely dependent on aviation, and air travel is sometimes the only way to reach certain parts of the archipelago. Based on previous research evidence, we speculated that air travel provided a path for the relocation diffusion of the B.1.466.2 variant from Java and Sumatra to the remote Papua [89, 91]. In the socio-economic dimension, Java is the center of gravity of Indonesia, followed by Sumatra. These two regions in Western Indonesia contribute about 80% of the national economy and interact frequently with convenient transportation conditions [90]. The phylogeographic reconstruction suggested that Java and Sumatra successively acted as epi-centers and formed stable transmission loops between them, reflecting the significant influence of the spatial structure of socio-economic factors on the diffusion pattern of the pandemic [91, 92].

Targeting an indigenous SARS-CoV-2 variant (B.1.466.2) circulating in Indonesia, we identified its evolutionary characteristics and transmission patterns by retrospective genomic surveillance. Although an epidemiological paradigm that combines molecular and native perspectives was provided, unevenly distributed and relatively inadequate genomic samples may bias the analytic results to some extent. In fact, sampling bias is inevitable in genomic surveillance and related research [93]. Therefore, in the process of deriving conclusions, we should try to combine the real-world pandemic situations and be cautious. As Indonesia improves the use of WGS for SARS-CoV-2 and strengthens international health cooperation, more accurate and real-time genomic surveillance for this variant and even comparative genomic analysis along with other variants are expected in the future.

## Conclusions

The SARS-CoV-2 lineage B.1.466.2 is considered to be an endemic variant and preceded the Delta variant as the dominant strain in Indonesia. This variant circulating in Indonesia was estimated to have emerged in Java Island since mid-June 2020 and to have evolved into two disproportional and distinct clades as of the end of August 2021. Most of the identified high-frequency non-synonymous mutations were found in the NSP3 and the spike protein, and the D614G/N439K/P681R co-mutations in the spike protein were observed. A four-phased trajectory of Ne was inferred, with an exponential growth from October 2020 to February 2021. The Rt was estimated to reach its peak in late December 2020 and to be less than one after early May 2021. Java and Sumatra successively acted as epi-centers of the Indonesian SARS-CoV-2 B.1.466.2 variant and formed stable transmission loops between them. A long-distance transmission link between the westernmost Sumatra and the easternmost Papua was inferred.

To our knowledge, this is the first comprehensive analysis of the genomic surveillance data of SARS-CoV-2 lineage B.1.466.2 in Indonesia, the world's largest archipelagic country. Our findings suggest that SARS-CoV-2 variants circulating in the tropical archipelago may follow unique patterns of evolution and transmission. Thus, continuous genomic surveillance with wider coverage and higher resolution is essential for the development of practicable and effective pandemic intervention strategies.

## Supplementary Information

**Additional file1** Acknowledgment Table for GISAID listing GISAID IDs, Originating Labs, Submitting Labs and Authors.

**Additional file2** Root-to-tip regression plot based on the maximum-likelihood phylogeny of SARS-CoV-2 lineage B.1.466.2 in Indonesia.

**Additional file3** Inferred transmission frequencies of SARS-CoV-2 lineage B.1.466.2 among different Indonesian regions in each phase.

## Data Availability

The datasets supporting the conclusions of this article are included within the article and its additional files.

## Abbreviations

COVID-19: Coronavirus disease 2019
SARS-CoV-2: Severe acute respiratory syndrome coronavirus 2
WHO: World Health Organization
ASEAN: Association of Southeast Asian Nations
VOCs: Variants of concern
VOIs: Variants of interest
WGS: Whole-genome sequencing
GISAID: Global initiative on sharing avian influenza data
VUMs: Variants under monitoring
ML: Maximum likelihood
MRCA: Most recent common ancestor
SNVs: Single nucleotide variants
Ne: Effective population size
Rt: Effective reproduction number
95% CI: 95% Confidence interval
NSP3: Non-structural protein 3
RBD: Receptor binding domain
ACE2: Angiotensin-converting enzyme 2
PPKM (Indonesian): Community-level public activity restrictions
PSBB (Indonesian): Large-scale social restrictions
