# Supplementary figures and images for: Tracking the molecular evolution and transmission patterns of SARS-CoV-2 lineage B.1.466.2 in Indonesia based on genomic surveillance data
Source: Virol J. 2022 Jun 16;19:103. doi: 10.1186/s12985-022-01830-1 (PMC9202327; doi:10.1186/s12985-022-01830-1)

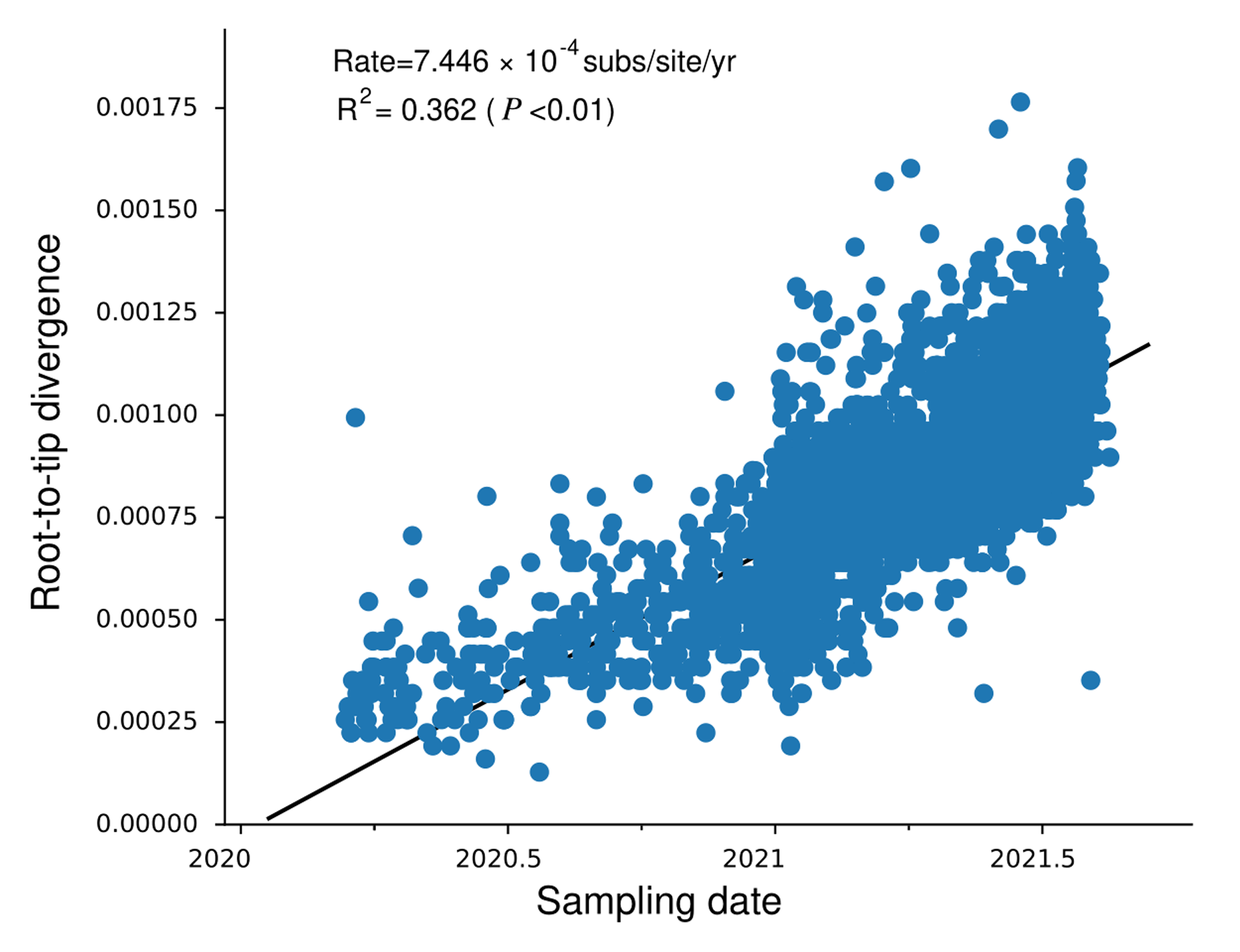

Supplement: Supplementary file 2 — Additional file2 Root-to-tip regression plot based on the maximum-likelihood phylogeny of SARS-CoV-2 lineage B.1.466.2 in Indonesia. [file 12985_2022_1830_MOESM2_ESM.tif]
