# Supplementary material for: Tracking the molecular evolution and transmission patterns of SARS-CoV-2 lineage B.1.466.2 in Indonesia based on genomic surveillance data
Source: Virol J. 2022 Jun 16;19:103. doi: 10.1186/s12985-022-01830-1 (PMC9202327; doi:10.1186/s12985-022-01830-1)
Supplement: Supplementary file 3 — Additional file3 Inferred transmission frequencies of SARS-CoV-2 lineage B.1.466.2 among different Indonesian regions in each phase. [file 12985_2022_1830_MOESM3_ESM.docx]

**Table S1.** Inferred transmission frequencies of SARS-CoV-2 lineage B.1.466.2 between Indonesian regions in each phase.

| Transmission event | | Inferred frequency |
| --- | --- | --- |
| **Phase Ⅰ (before 2020-09-27)** | | |
| *Java to Java* | | *6* |
| Java to Bali | | 2 |
| Java to Sumatra | | 1 |
| **Phase Ⅱ (2020-09-27 to 2021-02-28)** | | |
| *Java to Java* | | *286* |
| *Sumatra to Sumatra* | | *133* |
| *Bali to Bali* | | *80* |
| Java to Sumatra | | 35 |
| *Sulawesi to Sulawesi* | *28* | |
| Java to Kalimantan | 25 | |
| Java to Sulawesi | 19 | |
| Java to Bali | 16 | |
| *Kalimantan to Kalimantan* | *14* | |
| Sumatra to Java | 7 | |
| *Nusa-Tenggara to Nusa-Tenggara* | *6* | |
| Java to Nusa-Tenggara | 5 | |
| Sumatra to Kalimantan | 4 | |
| Java to Papua | 3 | |
| Sumatra to Papua | 3 | |
| Sumatra to Sulawesi | 3 | |
| Kalimantan to Bali | 2 | |
| Bali to Java | 1 | |
| Bali to Sulawesi | 1 | |
| Kalimantan to Nusa-Tenggara | 1 | |
| Nusa-Tenggara to Kalimantan | 1 | |
| Nusa-Tenggara to Papua | 1 | |
| Nusa-Tenggara to Sulawesi | 1 | |
| Nusa-Tenggara to Sumatra | 1 | |
| Sulawesi to Java | 1 | |
| Sulawesi to Kalimantan | 1 | |
| Sumatra to Nusa-Tenggara | 1 | |
| **Phase Ⅲ (2021-03-01 to 2021-05-06)** | | |
| *Sumatra to Sumatra* | *141* | |
| *Java to Java* | *26* | |
| *Bali to Bali* | *9* | |
| *Nusa-Tenggara to Nusa-Tenggara* | *6* | |
| *Kalimantan to Kalimantan* | *5* | |
| Sumatra to Kalimantan | 4 | |
| Nusa-Tenggara to Papua | 3 | |
| Bali to Java | 2 | |
| Kalimantan to Java | 2 | |
| *Papua to Papua* | *2* | |
| Sumatra to Nusa-Tenggara | 2 | |
| Java to Sumatra | 1 | |
| Papua to Kalimantan | 1 | |
| Sumatra to Java | 1 | |
| **Phase Ⅳ (after 2021-05-06)** | | |
| *Sumatra to Sumatra 43* | | |
| *Java to Java* | *24* | |
| *Kalimantan to Kalimantan* | *11* | |
| Java to Sumatra | 4 | |
| Nusa-Tenggara to Sulawesi | 3 | |
| Sumatra to Sulawesi | 3 | |
| Java to Kalimantan | 2 | |
| Sumatra to Java | 2 | |
| *Bali to Bali* | *1* | |
| Bali to Java | 1 | |
| Java to Sulawesi | 1 | |
| Kalimantan to Sumatra | 1 | |
| *Nusa-Tenggara to Nusa-Tenggara* | *1* | |
| *Sulawesi to Sulawesi* | *1* | |
| **Note:** The intra-regional transmission events and their inferred frequencies are shown in italics. | | |
